# Effects of a Chemically Characterised Multi-Component Nutraceutical Formulation on Intestinal, Hepatic and Skeletal Muscle Responses in an In Vitro Gut–Liver–Muscle Model

**DOI:** 10.3390/ijms27177759

**Published:** 2026-08-29

**Authors:** Rebecca Galla, Francesca Parini, Simone Mulè, Francesca Uberti

**Affiliations:** 1 Noivita s.r.l.s., Spin Off of University of Piemonte Orientale, Strada Privata Curti n. 7, 28100 Novara, Italy; rebecca.galla@noivita.it (R.G.); francescaparini00@gmail.com (F.P.); 2 Laboratory of Physiology, Department for Sustainable Development and Ecological Transition, University of Piemonte Orientale, UPO, 13100 Vercelli, Italy; simone.mule@uniupo.it

**Keywords:** autophagy, autophagic flux, gut–liver–muscle axis, non-alcoholic fatty liver disease, metabolic homeostasis, chemically characterised formulation, nutraceuticals

## Abstract

Autophagy plays a central role in cellular homeostasis and metabolic adaptation, and its dysregulation has been implicated in metabolic disorders, including non-alcoholic fatty liver disease (NAFLD). This study investigated the biological effects of a chemically characterised multi-component nutraceutical formulation using an integrated in vitro gut–liver–muscle axis model under lipotoxic and inflammatory conditions induced by free fatty acids (FFAs) and lipopolysaccharide (LPS). The principal bioactive constituents were quantified in both the individual extracts and the final formulation before biological testing. Caco-2, HepG2, and C2C12 cells were sequentially exposed to conditioned media to reproduce inter-organ metabolic interactions. The Supplement preserved intestinal barrier integrity by maintaining transepithelial electrical resistance and tight junction protein expression. In HepG2 cells, it preserved telomerase levels, improved markers of cellular metabolic adaptation, modulated AMPK/mTOR and SIRT1 signalling, and promoted autophagy-related responses, including increased LC3-II/I ratio, reduced p62 accumulation, and preservation of lysosomal markers. In skeletal muscle cells, exposure to conditioned medium derived from formulation-treated compartments was associated with improved cellular bioenergetics, reduced oxidative stress and inflammatory mediators, and enhanced ATP and glycogen levels under exercise-like conditions. Overall, these findings provide preliminary evidence that the chemically characterised formulation modulates interconnected pathways involved in intestinal barrier function, hepatic autophagy-related processes, and skeletal muscle metabolic adaptation under the experimental conditions employed.

## 1. Introduction

Autophagy is an evolutionarily conserved intracellular process that helps to maintain cellular homeostasis by eliminating damaged organelles, protein aggregates, and malfunctioning macromolecules via lysosomal recycling. This process is essential for cellular quality control and metabolic adaptation, especially under conditions of nutrient limitation, oxidative stress, or metabolic imbalance [1]. Autophagy is not only an essential housekeeping process but is also increasingly recognised as a critical mechanism involved in metabolic adaptation and cellular stress responses, with defective autophagic activity contributing to the pathogenesis of several metabolic disorders [2]. From a molecular point of view, autophagy is regulated by a complex network of nutrient- and energy-sensing signalling pathways that integrate environmental cues with cellular metabolic status [3]. Among these pathways, the mechanistic target of rapamycin (mTOR) pathway is considered a major negative regulator of autophagy. In nutrient-rich environments, mTOR complex 1 (mTORC1) is activated, promoting anabolic processes such as protein synthesis and cell growth while simultaneously suppressing autophagic activity [4]. In contrast, during states of energetic stress or nutrient deprivation, autophagy is activated by inhibition of mTOR signalling through the energy-sensing enzyme AMP-activated protein kinase (AMPK). This pathway is considered one of the major regulatory pathways of metabolism, cell growth, and autophagy [5]. Downstream of these signalling events, autophagy is mediated by a coordinated cascade of autophagy-related proteins (ATGs), including Beclin-1, Atg5, and LC3, which regulate autophagosome formation and maturation [6]. However, autophagosome accumulation alone is insufficient to demonstrate effective autophagic activity. Functional autophagic flux requires lysosomal degradation of cargo, involving p62 turnover and the preservation of lysosomal integrity, as indicated by markers such as lysosomal associated membrane proteins 1 and 2 (LAMP1, LAMP2), and Cathepsin D [7]. Pharmacological inhibitors such as chloroquine (CQ), which block lysosomal acidification, are commonly used to distinguish impaired degradation from true activation of autophagic recycling [8]. Autophagy is also closely linked to molecular pathways that regulate cellular bioenergetics and cellular metabolic adaptation. In particular, the sirtuin family of NAD^+^-dependent deacetylases act as metabolic sensors that coordinate energy metabolism, oxidative stress responses, and regulation of mitochondrial biogenesis [9]. Sirtuin-mediated deacetylation events can stimulate autophagy and enhance stress resistance. The transcriptional coactivator peroxisome proliferator-activated receptor gamma coactivator 1-alpha (PGC-1α) is also a key regulator of markers associated with mitochondrial biogenesis and oxidative metabolism [10]. These pathways interact with epigenetic regulatory mechanisms, including histone acetyltransferase (HAT) activity [11]. HAT activity is known to modulate chromatin structure and gene expression in response to metabolic signals. All these interconnected regulatory pathways help to maintain metabolic homeostasis [12]. Among these, non-alcoholic fatty liver disease (NAFLD), currently also referred to as metabolic dysfunction-associated steatotic liver disease (MASLD), has been identified as one of the most common chronic liver disorders in humans, which is characterised by the accumulation of lipids in hepatocytes, oxidative stress, and metabolic dysregulation [13]. Recent studies have indicated that alterations in autophagy, especially in lipophagy, are implicated in the development of fatty liver disease by affecting the normal lipid droplet turnover and mitochondrial function [14,15]. In this context, modulation of autophagy-related signalling pathways has been proposed as a promising therapeutic strategy to improve metabolic homeostasis and support liver health under conditions associated with metabolic dysfunction [16,17]. In recent years, significant interest has focused on nutraceutical compounds that modulate autophagy-related and metabolic signalling pathways [18]. Certain bioactive molecules have been identified as affecting autophagy, mitochondrial metabolism, and oxidative stress responses by regulating various signalling pathways, including AMPK, mTOR (Mammalian Target of Rapamycin), and sirtuin-dependent signalling [19,20]. Among these, spermidine, a naturally occurring polyamine found in various food products, including wheat germ, has been shown to extensively regulate autophagy and modulate metabolic homeostasis in several experimental models [21]. Similarly, another bioactive compound, polydatin, a precursor to resveratrol, has been shown to exert antioxidant and metabolic regulatory effects through various signalling pathways, including activation of AMPK and regulation of cellular bioenergetics [22]. Cellular bioenergetics is also strongly influenced by redox cofactors such as nicotinamide adenine dinucleotide (NADH). This compound plays a major role in mitochondrial respiration and the production of metabolic energy [23]. Furthermore, extracts of *Griffonia simplicifolia*, which contain 5-hydroxytryptophan (5-HTP), have been shown to significantly affect metabolic and neuroendocrine regulation. Although these bioactive compounds have individually been associated with modulation of metabolic and autophagy-related pathways, much less is known about the biological behaviour of chemically characterised multi-component formulations containing these constituents. On this basis, the biological evaluation of formulations containing multiple characterised constituents may provide information on their overall activity across interconnected metabolic pathways [24,25].

Based on this evidence, the objective of the present study was not to determine the biological contribution of individual constituents, but to evaluate the overall biological behaviour of a chemically characterised multi-component formulation following sequential exposure across intestinal, hepatic and skeletal muscle compartments. To effectively mimic the multi-organ crosstalk characteristic of metabolic and inflammatory stress, an integrated sequential setup combining intestinal (Caco-2), hepatic (HepG2), and skeletal muscle (C2C12) cell lines was employed. This multi-tissue approach allows for evaluating the systemic biological impact of the formulation along the gut–liver–muscle axis beyond a single isolated compartment. Using a sequential conditioned-medium in vitro model designed to reproduce selected aspects of intestinal–hepatic–muscle communication, we assessed the Supplement’s impact on intestinal permeability and epithelial barrier function; hepatic metabolic responses under conditions that simulate NAFLD; and skeletal muscle metabolic responses to investigate its effects on interconnected pathways implicated in NAFLD-associated metabolic dysfunction.

## 2. Results

### 2.1. Quantification of Selected Bioactive Constituents in the Individual Extracts and Final Formulation

The principal bioactive constituents of the botanical extracts used in the formulation were quantified to provide an analytical characterisation of the tested material.

Initially, the polydatin content in the *Fallopia japonica* extract was determined by HPLC. As shown in Table 1, the polydatin content was 98% of the total weight of the dry extract.

Subsequently, the wheat germ extract was examined for spermidine; similarly, the data presented in Table 1 indicate a content of 0.2%.

As shown in Table 1, vitamin C in the *Rosa canina* extract and 5-HTP in the *Griffonia simplicifolia* seed extract were also assessed. The measured contents were 70% and 25% of the respective dry extract weights.

Consequently, the selected marker compounds were subsequently quantified in the final formulation to verify whether their analytical levels remained constant after incorporation into the multicomponent mixture. As demonstrated in Table 2, the measured values were comparable to those obtained in the corresponding individual extracts, indicating that no substantial loss of the selected marker compounds occurred following formulation preparation.

However, it is important to note that these results should not be interpreted as the percentage composition of the final formulation. Instead, they should be interpreted as the analytical recovery of each selected marker compared to its expected content in the corresponding standardized extract

### 2.2. Impact of Supplement Treatment on Intestinal Function in NAFLD

**Figure 1:**
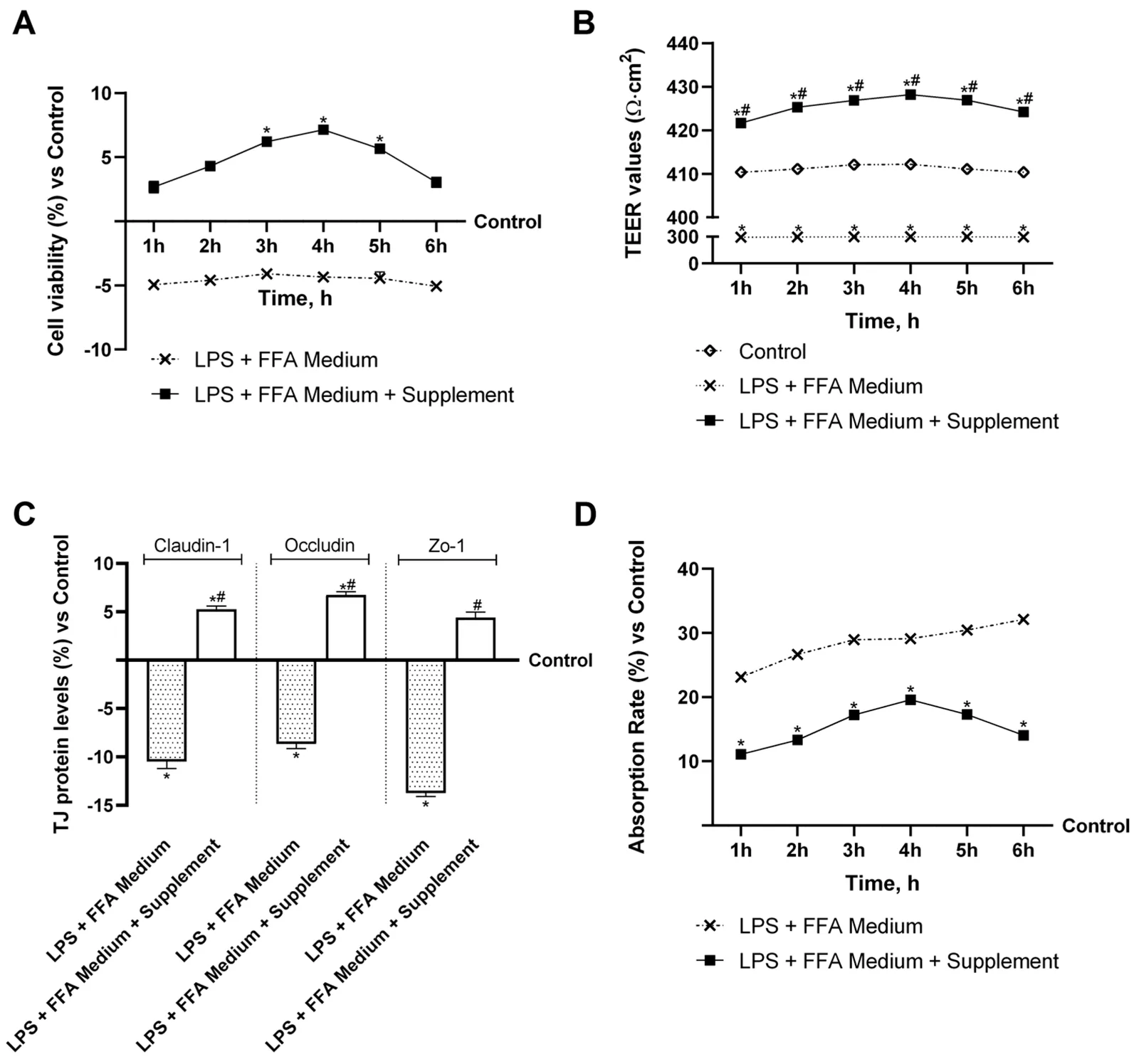
Intestinal safety analysis. In (**A**), cell viability; in (**B**), TEER values; in (**C**), TJ protein levels; and in (**D**), the percentage of paracellular permeability profile was determined using fluorescein as a tracer molecule to evaluate epithelial permeability and calculate Jmax. Data are expressed as mean ± SD (%) from five independent experiments, normalised to the control. LPS = 100 ng/mL; FFA = a mixture of palmitic acid and oleic acid. In (**A**,**D**), *p* < 0.05 vs. control (0% line); * *p* < 0.05 vs. LPS+FFA medium. In (**B**,**C**), * *p* < 0.05 vs. control; # *p* < 0.05 vs. LPS+FFA medium.

To investigate potential cytotoxic effects under metabolic stress, cell viability was first assessed in a lipotoxic and pro-inflammatory environment induced by LPS and free fatty acids [26]. As illustrated in Figure 1A, Supplement treatment reversed the LPS+FFA-induced decline in viability, restoring values towards levels comparable to the untreated control (*p* < 0.05). The cytoprotective effect was most evident at 4 h, with significant recovery compared to both control and damaged conditions (*p* < 0.05). These observations were supported by transepithelial electrical resistance (TEER) measurements and TJ protein analysis (Figure 1B), showing that the Supplement preserved monolayer stability throughout the experiment. TEER values remained significantly higher in Supplement-treated cells than in the LPS+FFA group, increasing by approximately 43% at 4 h (*p* < 0.05). At the molecular level, the expression of key TJ proteins, such as claudin, occludin, and ZO-1, was markedly increased (Figure 1C). Supplement treatment resulted in about a 200% increase in claudin, a 128% increase in occludin, and a 313% increase in ZO-1 compared to the LPS+FFA condition (*p* < 0.05). While ELISA quantifies total protein levels without resolving spatial subcellular localisation, the concurrent preservation of high TEER values strongly supports the functional assembly and membrane anchoring of these junctional proteins. This effect was reflected in the paracellular permeability profile determined using fluorescein as a tracer molecule (Figure 1D): while LPS+FFA-induced damage led to a progressive increase in permeability (*p* < 0.05), the Supplement partially restored the permeability values to levels similar to those observed under control conditions, with a transient peak at 4 h (*p* < 0.05).

### 2.3. Impact of Supplement Treatment on Hepatic Autophagy in NAFLD

**Figure 2:**
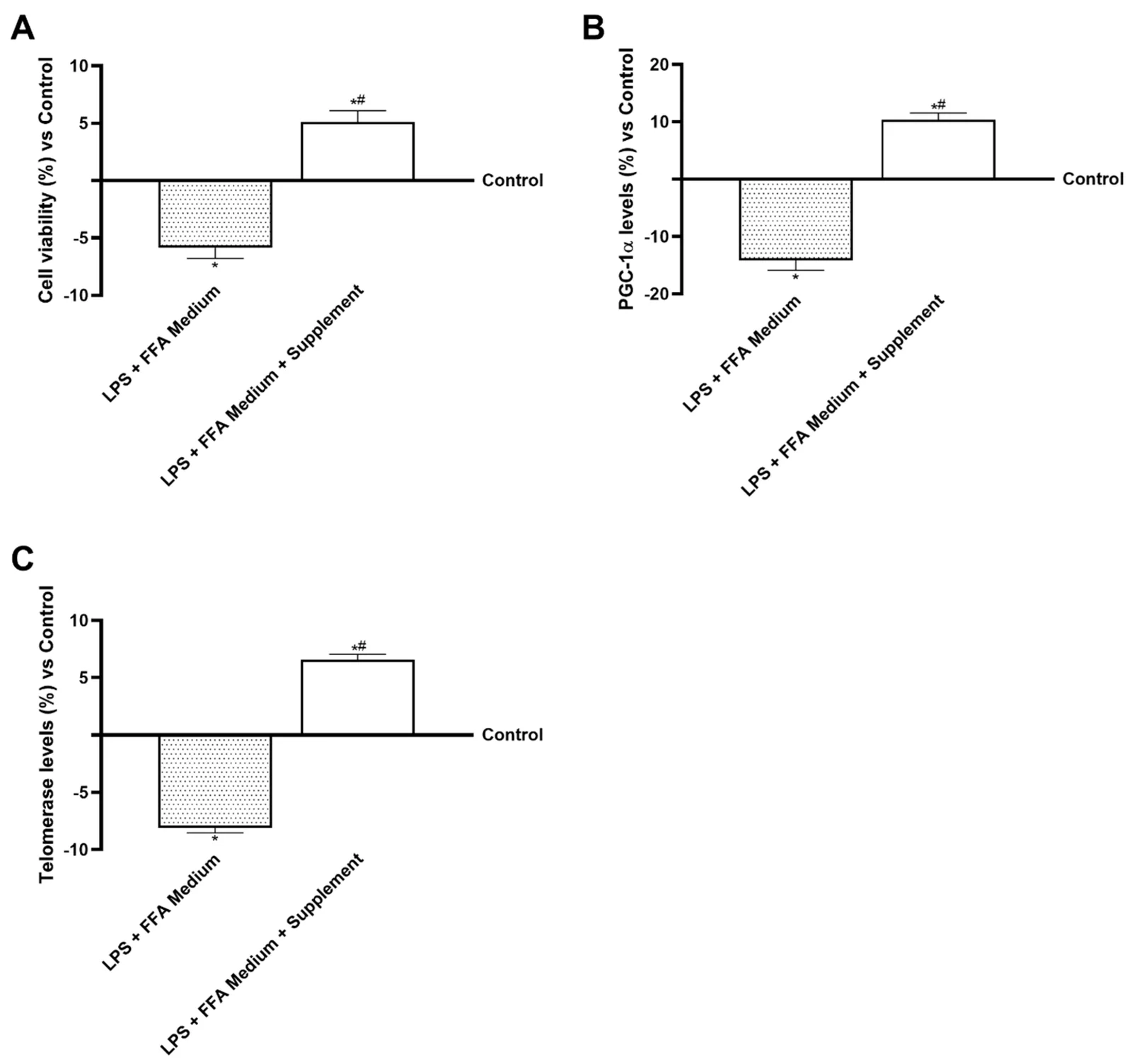
Assessment of cellular and metabolic stability. In (**A**), cell viability; in (**B**), PGC-1α levels; and in (**C**), telomerase levels were assessed using a specific ELISA kit. Data are expressed as mean ± SD (%) from five independent experiments and are normalised to the control. * *p* < 0.05 vs. control; # *p* < 0.05 vs. LPS+FFA medium.

To assess the biological effects of the formulation following intestinal processing, we examined cellular and metabolic stability by monitoring cell viability, PGC-1α levels, and telomerase activity. As illustrated in Figure 2A, exposure to the LPS+FFA medium induced a slight decline in cell viability compared to untreated controls (*p* < 0.05). Notably, the Supplement effectively reversed this trend, not only restoring viability relative to the LPS+FFA challenge (about 115% higher vs. LPS+FFA, *p* < 0.05) but bringing values back to levels comparable to, or even slightly exceeding, the control (*p* < 0.05). We then focused on mitochondrial biogenesis (PGC-1α) and telomerase activity as key markers of metabolic resilience and stress response. Data presented in Figure 2B,C show that LPS+FFA-induced damage significantly suppressed both PGC-1α and telomerase levels compared to the control (*p* < 0.05). Consistent with the cell viability results, the Supplement treatment successfully counteracted this decline, boosting PGC-1α and telomerase levels by 137% and 123%, respectively, compared to the damaged state (*p* < 0.05). Taken together, these findings suggest an improvement in cellular metabolic adaptation under lipotoxic conditions.

**Figure 3:**
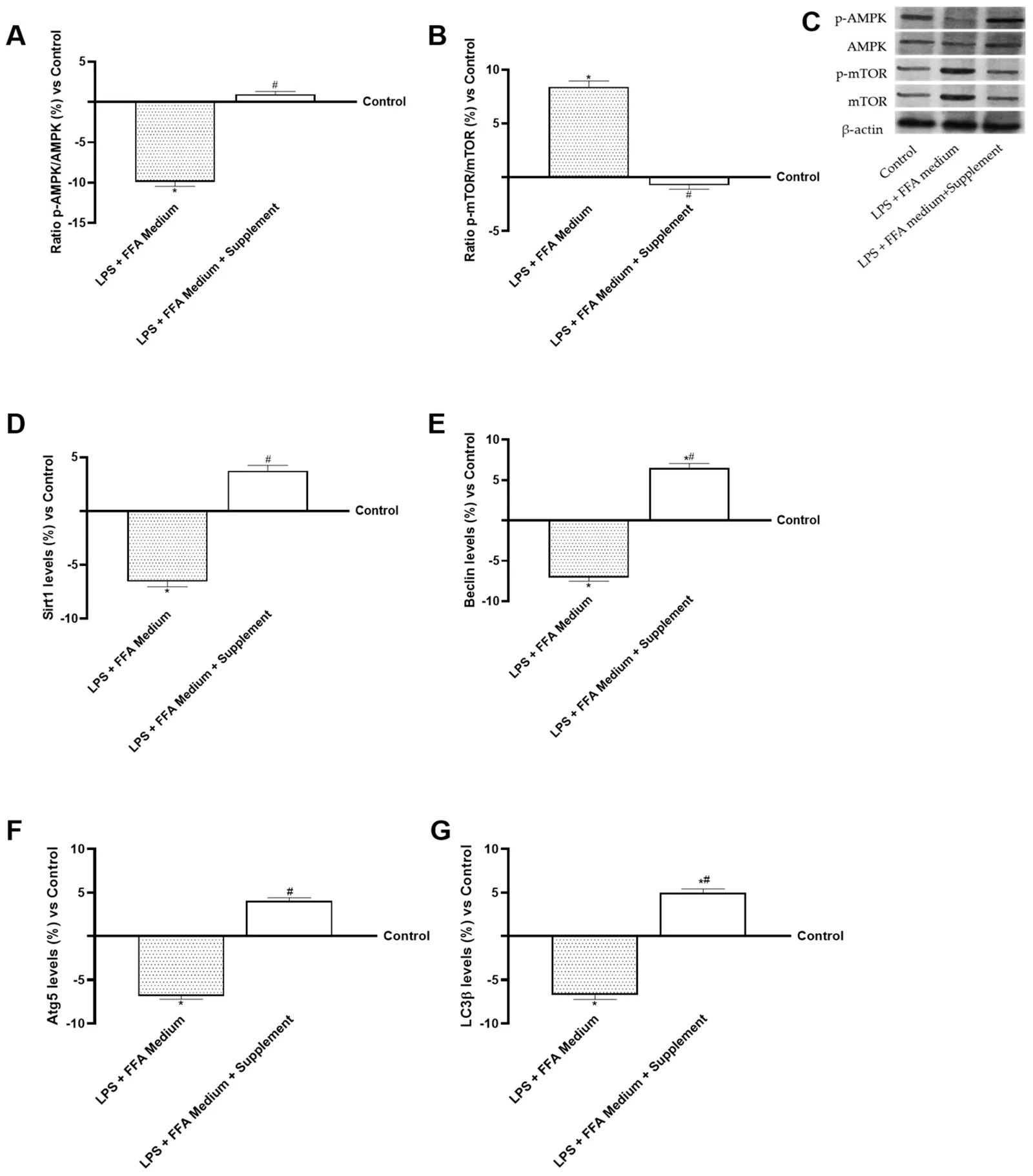
Assessment of hepatic nutrient sensing and autophagy markers in HepG2 cells. (**A**) p-AMPK/AMPK ratio and (**B**) p-mTOR/mTOR ratio; (**C**) representative Western blot bands for p-AMPK, AMPK, p-mTOR, and mTOR; (**D**) SIRT1 levels; (**E**) Beclin-1 levels; (**F**) Atg5 levels; and (**G**) LC3β levels. Regarding Atg5 modulation, HAT levels were also assessed and are reported in Appendix A, Figure 1. Data are expressed as the mean ± SD (%) of five independent experiments and normalised to the control. * *p* < 0.05 vs. control; # *p* < 0.05 vs. LPS+FFA medium.

**Figure A1:**
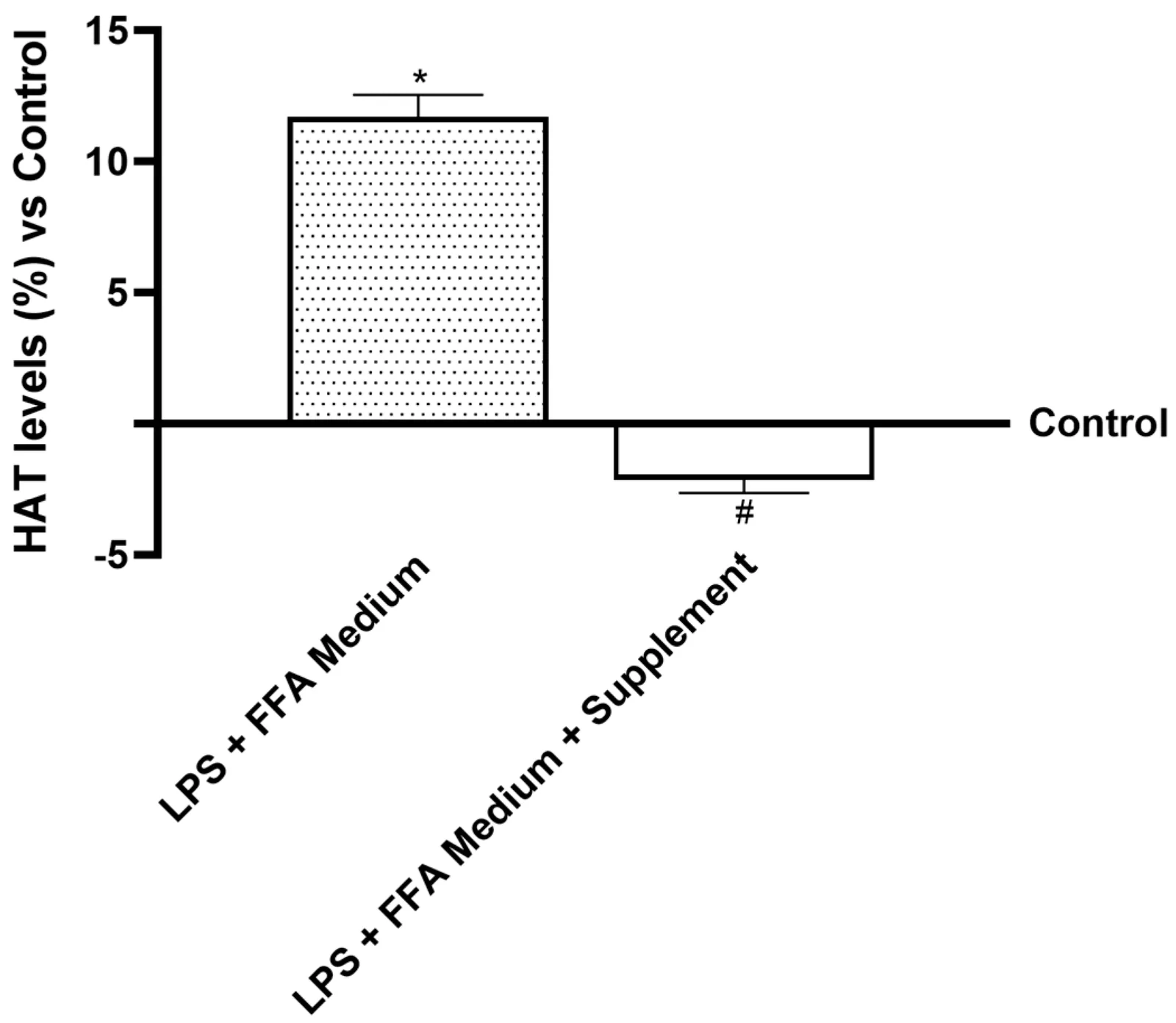
HAT levels in cells treated with LPS+FFA Medium and Supplement. In the graph, HAT levels were assessed using an ELISA kit. Data are expressed as mean ± SD (%) of five independent experiments and normalised to the control. The horizontal baseline represents the untreated control. * *p* < 0.05 vs. control; # *p* < 0.05 vs. LPS+FFA medium.

To further investigate the molecular responses associated with formulation treatment in HepG2 cells, key nutrient-sensing and autophagic pathways were modulated (Figure 3). As illustrated in Figure 3A,B and supported by the representative Western blot bands shown in Figure 3C, the exposure to LPS+FFA medium significantly disrupted the energy-sensing balance, characterized by a marked reduction in the p-AMPK/AMPK (*p* < 0.05) ratio and a concomitant increase in the p-mTOR/mTOR ratio (*p* < 0.05), indicating a suppression of metabolic catabolism in favour of protein synthesis. Formulation treatment significantly modulated this stress-induced profile. Compared with the damaged LPS+FFA cells, the Supplement increased the p-AMPK/AMPK ratio by approximately 12% (*p* < 0.05), not only recovering the loss but also slightly exceeding baseline control levels. Simultaneously, formulation treatment significantly reduced the increase in the p-mTOR/mTOR ratio induced by the lipotoxic stimulus. This response was accompanied by a significant recovery of SIRT1 levels (Figure 3D). While LPS+FFA treatment suppressed this energy sensor, the addition of the Supplement increased SIRT1 expression by more than 10% relative to the damaged state (*p* < 0.05), effectively surpassing levels in untreated cells. Consistent with SIRT1/AMPK activation, markers associated with autophagic flux showed a significant recovery (Figure 3E–G). Although the LPS+FFA challenge inhibited the expression of Beclin-1, Atg5, and LC3β, the Supplement strongly counteracted this inhibition. Specifically, compared with the damaged state, the Supplement increased Beclin-1 by about 14%, Atg5 by about 11%, and LC3β by about 12% (*p* < 0.05). In relation to the modulation of Atg5 and the epigenetic control of these pathways, HAT levels were also evaluated; as reported in Appendix A, Figure A1, these levels exhibited a consistent trend, further supporting the Supplement’s role in coordinating the cellular response to metabolic stress. Taken together, these results indicate that formulation treatment modulated the AMPK/SIRT1 axis and several proteins associated with autophagosome formation and autophagic recycling under the experimental stress conditions.

**Figure 4:**
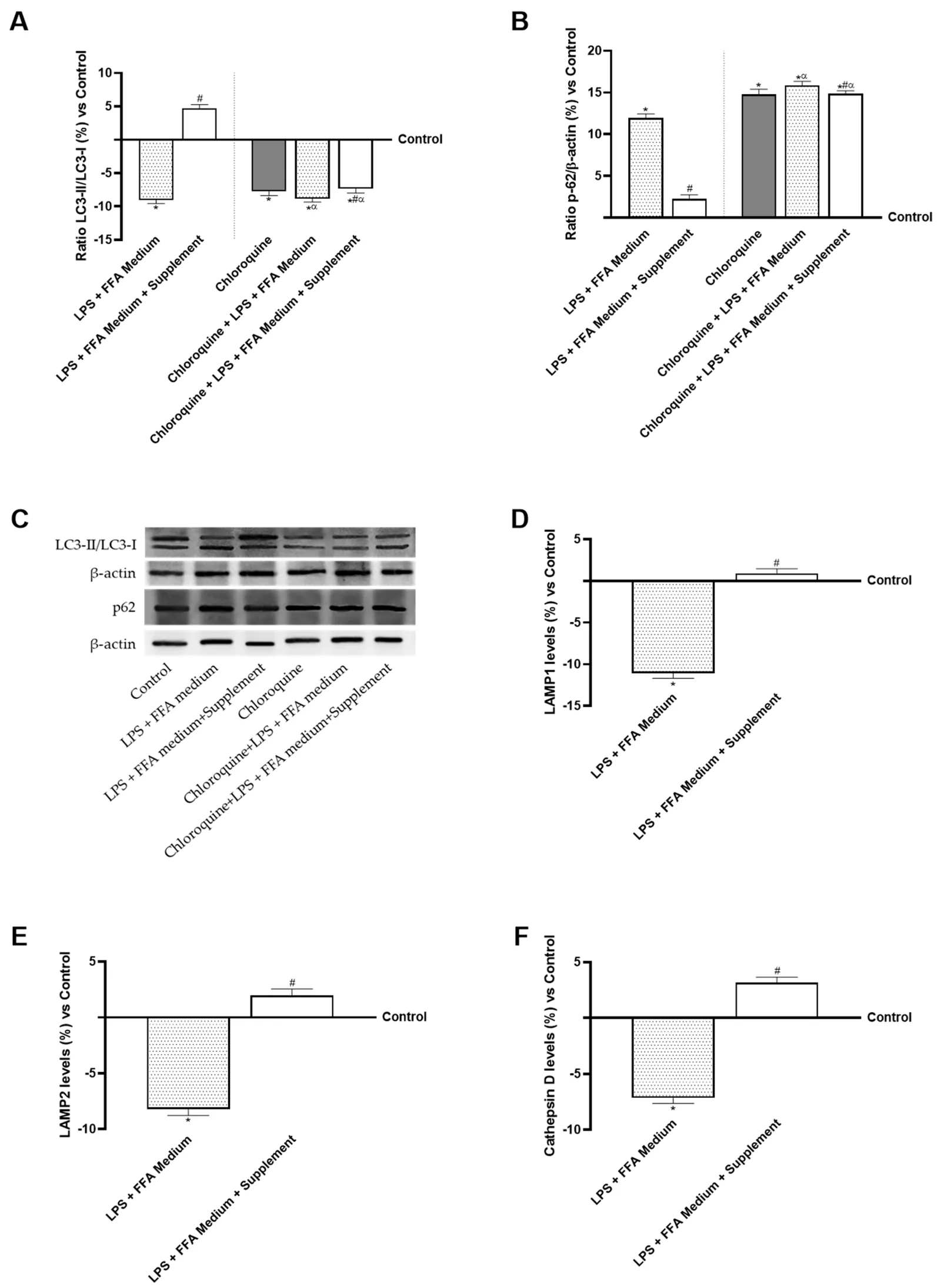
Assessment of hepatic autophagy flux and lysosomal markers in HepG2 cells. (**A**) Ratio of LC3-II/LC3-I; (**B**) ratio of p62/β-actin; (**C**) representative Western blot bands for LC3-II/LC3-I, p62, and β-actin (used as a loading control); (**D**) LAMP1 levels; (**E**) LAMP2 levels; and (**F**) Cathepsin D levels. The ratios (**A**,**B**) were determined by Western blot analysis, while levels of lysosomal markers (**D**–**F**) were measured using specific ELISA kits. Data are expressed as mean ± SD (%) of five independent experiments and normalised to the control. * *p* < 0.05 vs. control; # *p* < 0.05 vs. LPS+FFA medium; α *p* < 0.05 vs. CQ.

Following the analysis of initiation markers, the completion of the autophagic flux was assessed by monitoring the conversion of LC3-I to LC3-II and the degradation of the cargo protein p62. As illustrated in Figure 4A and supported by the representative Western blot lanes in Figure 4C, the LC3-II/LC3-I ratio significantly decreased following LPS+FFA-induced damage (*p* < 0.05). Supplement treatment effectively reversed this inhibition, increasing the ratio by approximately 50% compared to the damaged condition (*p* < 0.05). To further investigate the potential association of the observed changes in LC3-II/LC3-I and p62 with lysosome-dependent autophagy-related turnover, experiments were conducted in the presence of CQ. The addition of CQ resulted in the abolition of the increase in the LC3-II/LC3-I ratio associated with the Supplement, indicating that the observed response was dependent on lysosomal function. Parallel results were obtained for p62 levels (Figure 4B). Exposure to the lipotoxic medium led to an accumulation of p62 (+11.8% vs. control, *p* < 0.05), indicating a stagnation of the autophagic flux. As demonstrated in the results, the administration of the supplement resulted in a nearly 10% reduction in p62 accumulation in comparison with the damaged state (*p* < 0.05). Again, the presence of CQ eliminated this protective effect, resulting in a marked accumulation of p62 regardless of treatment. To further evaluate total lysosomal marker abundance, overall protein levels of key lysosomal components were assessed via ELISA (Figure 4D–F). LPS+FFA exposure significantly suppressed the total expression of LAMP1, LAMP2, and Cathepsin D. Supplement treatment significantly attenuated this suppression, boosting the total protein levels of these markers by approximately 11–13% compared to the LPS+FFA-damaged cells (*p* < 0.05) and restoring them to near-control values. Collectively, these findings indicate that the Supplement modulates autophagy-related pathways and is associated with improved lysosome-dependent protein turnover and preservation of lysosomal markers under metabolic stress conditions.

Although the present findings are consistent with improved autophagy-related turnover, the use of pharmacological inhibitors and protein markers does not entirely rule out the contribution of parallel degradation pathways. Further studies using genetic approaches will be required to confirm the causal involvement of specific signalling nodes.

### 2.4. Impact of Supplement Treatment on Skeletal Muscle Function

**Figure 5:**
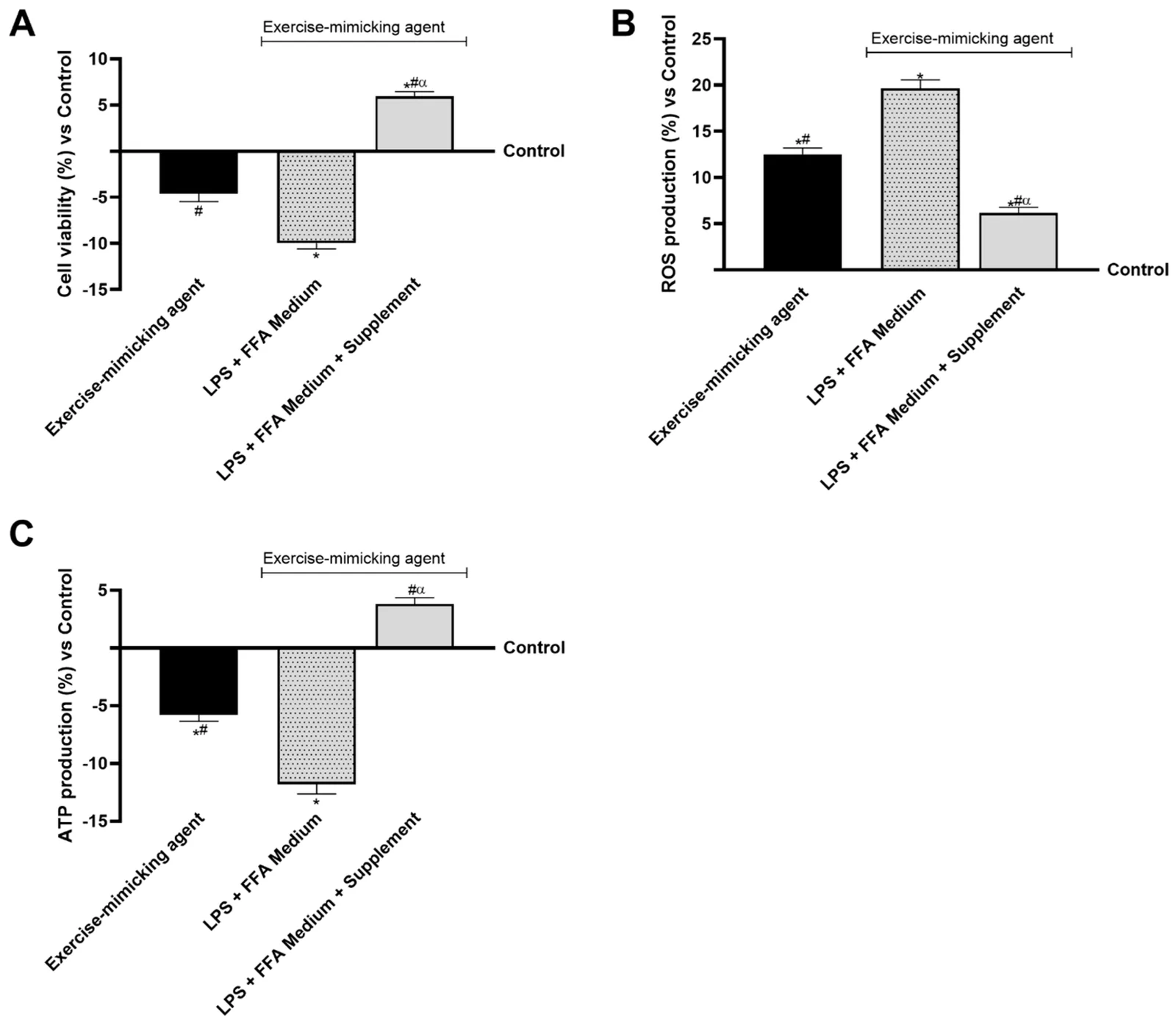
Assessment of cellular stability and redox balance. In (**A**), cell viability; in (**B**), ROS production; and in (**C**), ATP production. Data are expressed as mean ± SD (%) from five independent experiments and are normalised to the control. * *p* < 0.05 vs. control; # *p* < 0.05 vs. LPS+FFA medium; α *p* < 0.05 vs. exercise stimulus.

A lipotoxic and pro-inflammatory metabolic stress condition can significantly affect muscle metabolism. Through the liver–muscle axis, hepatic alterations may impair skeletal muscle’s ability to regulate energy metabolism, leading to reduced glycogen synthesis and increased oxidative stress. For this reason, the muscle model was included to assess whether the Supplement could counteract not only localised organ damage but also peripheral metabolic complications and alterations in muscle energy metabolism associated with chronic metabolic stress. Muscle cells were subjected to a functional stimulation protocol consisting of a 1 h pre-treatment with caffeine to mimic exercise-like conditions. Cell viability was first evaluated following exposure to conditioned medium collected from the hepatic compartment under these stimulated conditions. As shown in Figure 5A, LPS+FFA-induced damage reduced cell viability compared to both the untreated control and the exercise stimulus (exercise-mimicking agent) alone (*p* < 0.05). In contrast, the metabolite derived from Supplement treatment counteracted this effect, increasing cell viability by 167% relative to the damaged LPS+FFA condition (*p* < 0.05). Notably, the formulation-conditioned group also showed higher values than the exercise-mimicking stimulus alone (77% increase; *p* < 0.05), suggesting an additional protective effect under the experimental conditions. A similar pattern was observed for ROS production and ATP levels (Figure 5B,C). LPS+FFA exposure led to a significant increase in oxidative stress and a simultaneous drop in ATP production (*p* < 0.05), indicating impaired energy metabolism. Formulation-conditioned medium significantly modified this metabolic profile: compared with the damaged condition, ROS production was reduced by about 69% (*p* < 0.05). In parallel, ATP availability showed a marked recovery. The Supplement increased ATP levels by about 311% compared to the LPS+FFA condition (*p* < 0.05), and by 153% compared to the exercise stimulus alone.

**Figure 6:**
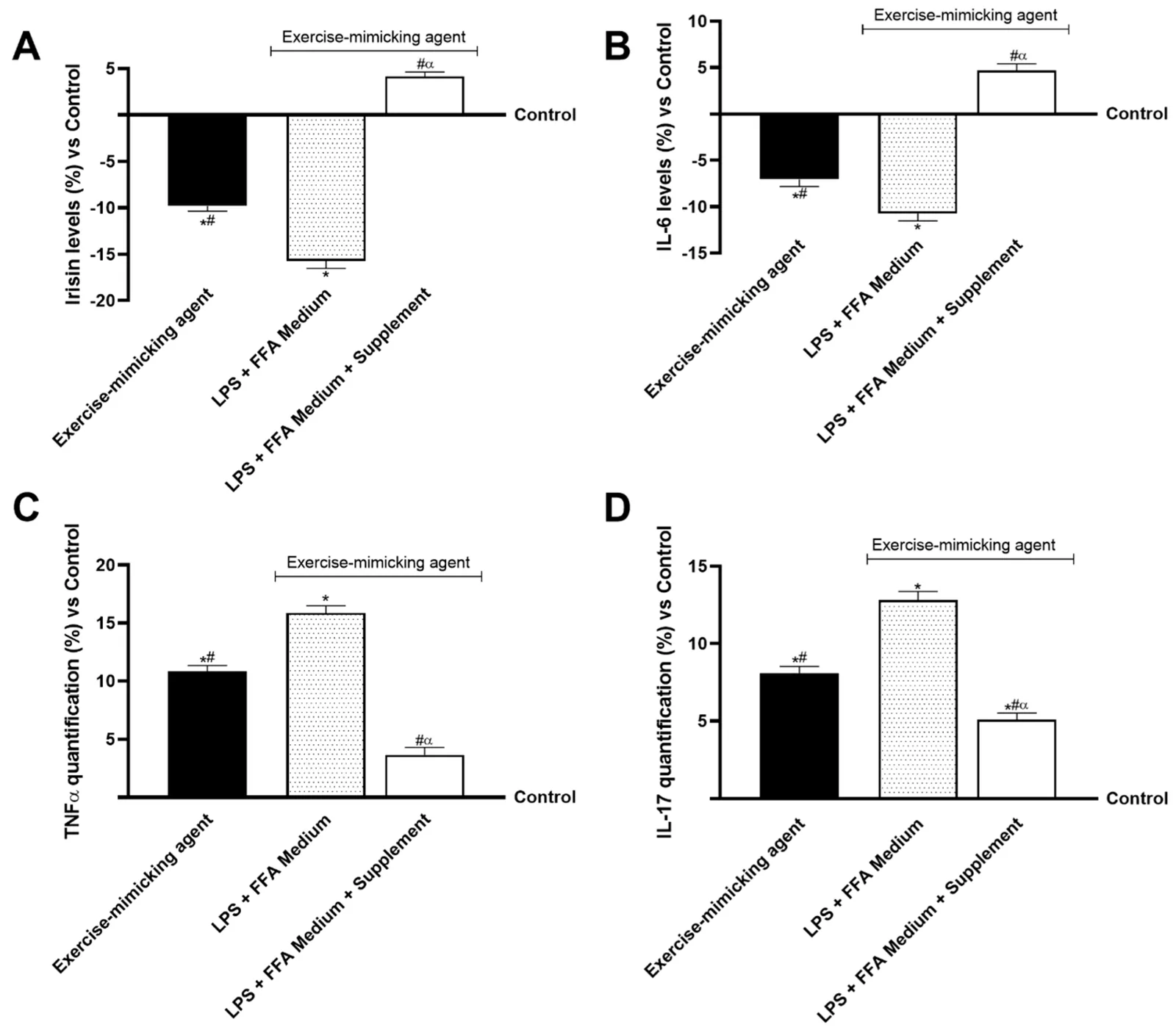
Analysis of myokine and cytokine levels. In (**A**), irisin levels; in (**B**), IL-6 levels; in (**C**), TNF-α levels; and in (**D**), IL-17 levels. Data are expressed as mean ± SD (%) of five independent experiments and normalised to the control. * *p* < 0.05 vs. control; # *p* < 0.05 vs. LPS+FFA medium; α *p* < 0.05 vs. exercise stimulus.

To assess the response to lipotoxic stress and the effectiveness of treatment, the balance between pro-inflammatory signals and protective factors was examined. Specifically, measuring TNF-α and IL-17 indicated the extent of the pro-inflammatory response associated with metabolic damage. Simultaneously, analysing myokines such as IL-6 and irisin released during exercise helped assess the tissue’s ability to initiate adaptive responses, linking energy metabolism to the resolution of inflammation. As shown in Figure 6A–D, exposure to LPS+FFA resulted in decreased irisin and IL-6, while increasing TNF-α and IL-17 compared to controls (*p* < 0.05). Supplement treatment notably modulated these effects, increasing irisin and IL-6 levels by approximately 380% and 229%, respectively (*p* < 0.05), whereas TNF-α and IL-17 levels were reduced by approximately 77% and 60%, respectively (*p* < 0.05), compared with the damaged LPS+FFA condition. In this context, IL-6 modulation should be interpreted as part of an adaptive myokine response associated with exercise-like metabolic stimulation rather than as a marker of systemic inflammation. Even relative to the exercise stimulus, the conditioned medium derived from the formulation-treated hepatic compartment was associated with lower cytokine levels than the exercise stimulus alone under the experimental conditions employed.

**Figure 7:**
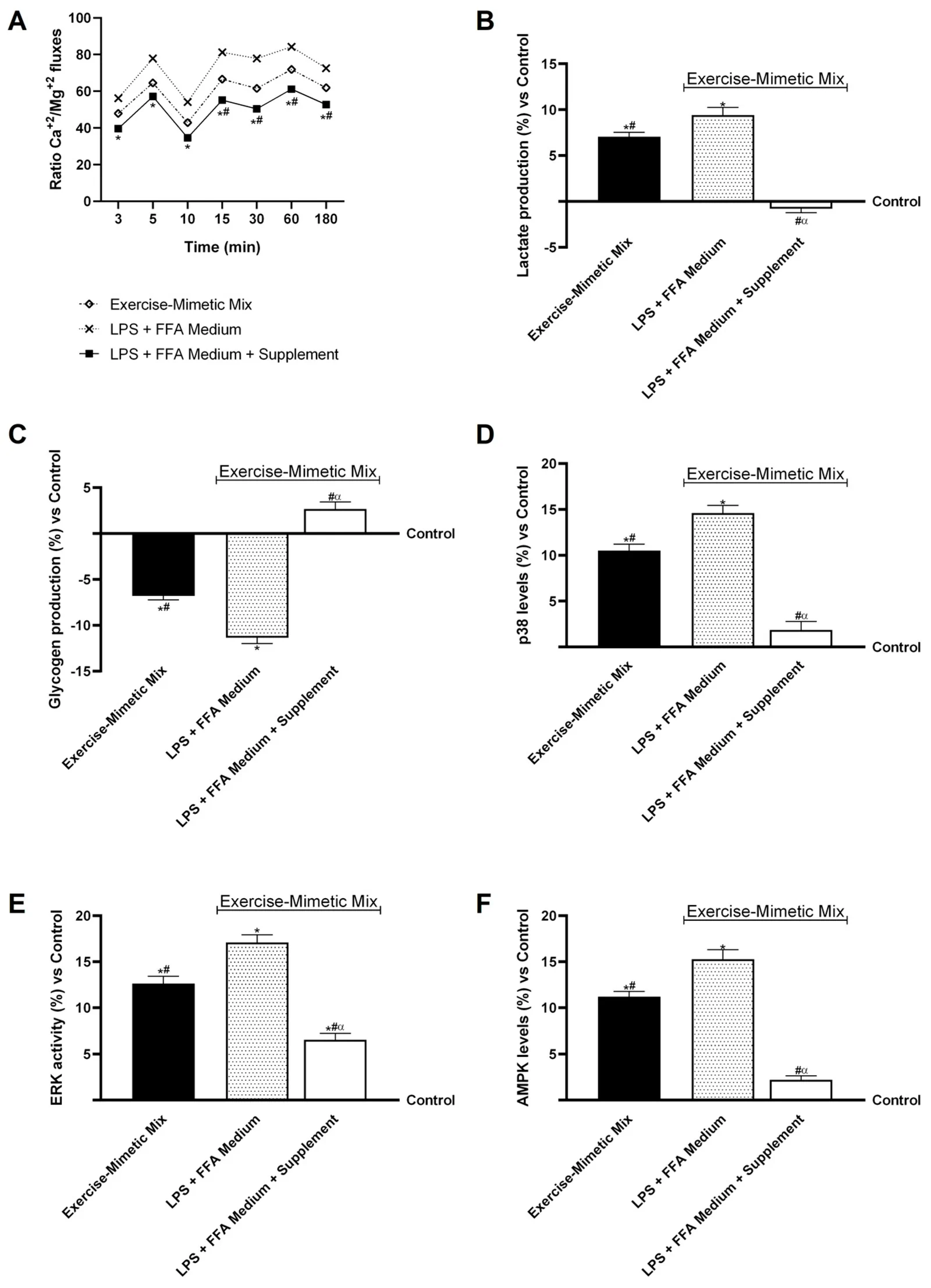
Assessment of the response to exercise conditions. In (**A**), calcium and magnesium fluxes evaluated using Fura-2-AM and Mag-Fura-2-AM (Furaptra) probes; in (**B**), lactate production; in (**C**), glycogen production; in (**D**) p-p38 levels; in (**E**), p-ERK levels; and in (**F**), p-AMPK levels. Data are expressed as mean ± SD (%) of five independent experiments and normalised to the control. * *p* < 0.05 vs. control; # *p* < 0.05 vs. LPS+FFA medium; α *p* < 0.05 vs. exercise stimulus.

Subsequently, under simulated exercise conditions, markers of contractile dynamics and cellular energy metabolism were evaluated. In particular, calcium and magnesium fluxes, together with lactate production and intracellular glycogen levels, were analysed as indicators of the muscle cell’s ability to sustain energetic demand (Figure 7). As shown in Figure 7A, LPS+FFA exposure resulted in a sustained increase in the Ca^2+^/Mg^2+^ ratio throughout the entire time course relative to both the control and the exercise stimulus (*p* < 0.05), indicating an imbalance in ionic homeostasis and altered calcium handling. In contrast, Supplement treatment partially restored the Ca^2+^/Mg^2+^ ratio toward physiological values (*p* < 0.05), particularly at 30, 60, and 180 min, suggesting improved contractile stability. A similar trend was observed for lactate levels (Figure 7B). Both the exercise stimulus and LPS+FFA treatment increased lactate production compared to control (*p* < 0.05), reflecting a shift toward anaerobic glycolysis. However, Supplement treatment significantly reduced lactate levels compared with the LPS+FFA condition (approximately 1180% reduction, *p* < 0.05). In line with these findings, intracellular glycogen levels (Figure 7C) were markedly reduced in the presence of the exercise stimulus following LPS+FFA treatment compared to control (*p* < 0.05). Conversely, Supplement treatment significantly increased glycogen levels relative to the damaged condition (approximately 183%, *p* < 0.05), suggesting an improved capacity for energy storage and utilisation in muscle cells.

Finally, the mechanisms underlying muscle adaptation to energetic stress were investigated. Specifically, the AMPK, p38, and ERK signalling pathways represent key nodes involved in muscle adaptation to both energetic and functional stress. Proper activation of these pathways coordinates the restoration of homeostasis and enhances muscle fibre performance, thereby distinguishing a beneficial training stimulus from chronic stress, which can lead to protein degradation or mitochondrial dysfunction. As shown in Figure 7D, p38 levels were increased under LPS+FFA-induced damage during simulated exercise compared to both the untreated control (*p* < 0.05) and the exercise stimulus alone (*p* < 0.05). Similarly, ERK (Figure 7E) and AMPK (Figure 7F) levels were elevated under LPS+FFA conditions relative to the control (*p* < 0.05) and to the exercise stimulus (*p* < 0.05). In contrast, Supplement treatment effectively modulated these signalling pathways, restoring p38, ERK, and AMPK levels closer to control values and suggesting normalisation of the metabolic stress response (*p* < 0.05). Specifically, Supplement treatment reduced p38, ERK, and AMPK levels by about 87%, 62%, and 86%, respectively, compared to the damaged condition (*p* < 0.05). These results indicate that conditioned medium derived from formulation-treated compartments modulated stress-related signalling pathways towards values more comparable to those observed under control conditions.

## 3. Discussion

In recent years, increasing attention has been directed toward developing integrated approaches to evaluate the biological activity of nutraceutical compounds across multiple physiological systems. This shift reflects a growing recognition of the need to move beyond reductionist models and toward systems-level investigations that capture complex biological interactions, as highlighted by recent clinical and mechanistic studies [27,28]. In this context, the present study was designed to investigate the effects of a multi-component Supplement on autophagy-related pathways and metabolic homeostasis using an in vitro model that mimics the intestinal–hepatic–muscle axis, thereby modelling selected sequential interactions relevant to the intestinal–hepatic–muscle axis following oral exposure. The sequential conditioned-medium strategy adopted in the present work represents an established experimental approach for investigating selected aspects of inter-organ communication under controlled in vitro conditions. Rather than reproducing the complete physiological gut–liver–muscle axis, this model was specifically designed to investigate mechanistic responses generated sequentially between cellular compartments following intestinal processing. Similar approaches have been described for functionally coupled multi-organ systems and are recognised as valuable tools for studying intercompartmental signalling while preserving compartment-specific experimental conditions [29,30]. The Supplement exhibited a protective effect against metabolic stress induced by inflammatory and lipotoxic stimuli (LPS+FFA) in the intestine. The maintenance of epithelial barrier integrity was clearly demonstrated by the preservation of TEER values and the significant upregulation of key tight junction proteins, including claudin, occludin, and ZO-1. Although total protein quantification via ELISA does not directly evaluate spatial subcellular localisation or membrane assembly, the sustained TEER values strongly support functional barrier preservation and effective membrane anchoring. It should be noted that fluorescein permeation was employed as a functional marker of epithelial permeability and absorptive behaviour of the intestinal barrier rather than as a direct measurement of the transport of formulation-derived constituents. Therefore, the observed effects should be interpreted as changes in barrier function rather than direct evidence of intestinal absorption of the tested formulation. These results suggest that intestinal barrier modulation may be an upstream mechanism that helps preserve metabolic homeostasis, given the pivotal role of intestinal permeability in the development of systemic metabolic dysfunction. Furthermore, the Supplement’s ability to modulate permeability kinetics and preserve epithelial integrity is supported by its capacity to maintain cellular viability and regulate permeability, thereby limiting the increase in paracellular permeability induced by the experimental lipotoxic and inflammatory challenge. This is consistent with previous studies demonstrating that preservation of intestinal barrier integrity is a key determinant of metabolic homeostasis and systemic inflammation [31,32]. The hepatic model demonstrated that the Supplement mitigated the negative effects of a high-glucose, lipotoxic milieu following intestinal processing. While the HepG2-based model reproduces key features of hepatic metabolic and lipotoxic stress, it does not fully capture the multi-stage complexity of NAFLD pathophysiology, particularly chronic lipid droplet accumulation, triglyceride storage, liver injury markers (such as AST/ALT), and multicellular interactions.

A strength of the present study is the analytical characterisation of the principal bioactive constituents before biological testing, together with verification of their stability in the final formulation. This improves the chemical definition and reproducibility of the experimental system compared with studies relying solely on label declarations.

The modulation of mitochondrial-associated markers and cellular adaptation is suggested by the recovery of cell viability and the rise in PGC-1α and telomerase levels. In line with previous reports, PGC-1α is widely recognised as a central regulator of mitochondrial biogenesis and cellular adaptation to metabolic stress, supporting the interpretation that metabolic adaptation is improved under these conditions [33]. These findings support the hypothesis that the Supplement may help maintain hepatic homeostasis under metabolic stress. This is particularly relevant given the liver’s central role in coordinating metabolic regulation and processing orally administered compounds [34]. At the molecular level, particular attention was paid to nutrient-sensing pathways that regulate autophagy and energy metabolism. The Supplement successfully mitigated the disruption of the SIRT1–mTOR axis observed under stress conditions by increasing SIRT1 levels and decreasing mTOR activity towards control values. The observed alterations in the AMPK/mTOR and SIRT1 pathways could suggest the initiation of adaptive metabolic responses. This balance is consistent with a cellular environment that supports processes linked to autophagy and metabolic adaptability. Indeed, the SIRT1–mTOR axis has been extensively described as a critical regulatory node that controls autophagy induction and cellular stress responses across various pathological contexts [35,36]. The intricate relationship between metabolic signalling and gene regulation is further highlighted by the modulation of HAT activity, suggesting the involvement of epigenetic mechanisms. In line with these observations [37], the Supplement modulated key proteins involved in autophagosome formation, including LC3β, Beclin-1, and Atg5. Importantly, the combined evaluation of the LC3-II/LC3-I ratio, p62 (SQSTM1) degradation, lysosomal markers, and CQ-mediated inhibition provided evidence consistent with the restoration of autophagy-related flux under metabolic stress conditions. In metabolic stress models, the accumulation of autophagosomes often results from impaired lysosomal clearance rather than from true activation of autophagy. The data demonstrated that the administration of the supplement resulted in an augmentation of the LC3-II/LC3-I ratio, concurrently accompanied by a diminution in p62 accumulation, in comparison with the condition that had sustained damage. These observations are frequently interpreted as being in accordance with the enhancement of autophagy-related turnover. Evidence for the involvement of lysosome-dependent processing was further obtained through the CQ challenge. The effects of the supplement on LC3-II/LC3-I and p62 were abolished by CQ-induced inhibition of lysosomal function, suggesting that these responses were dependent on a functional degradative compartment. This process is further supported by the restoration of lysosomal markers, including LAMP1, LAMP2, and mature Cathepsin D, suggesting preservation of lysosomal integrity and enzymatic activity, thereby alleviating the metabolic congestion induced by lipotoxic stress. However, according to the current autophagy guidelines [38], these findings support improved autophagy-related turnover, but do not constitute definitive proof of complete autophagic flux restoration. Further mechanistic studies should be conducted to confirm this interpretation. While ELISA measurements quantify overall protein abundance, future studies using immunofluorescence and confocal microscopy will be useful to evaluate changes in lysosomal subcellular localisation and morphology. These markers are widely used as indicators of autophagy pathway engagement and have been associated with cellular adaptation to stress conditions across multiple experimental models [39]. The skeletal muscle model provided additional information on downstream cellular responses to conditioned medium derived from the hepatic compartment. The use of different biological endpoints across Caco-2, HepG2, and C2C12 cells reflects the specific physiological role of each compartment within the sequential model. Consequently, the selection of biomarkers was based on their relevance to intestinal barrier function, hepatic metabolic and autophagy-related processes, or skeletal muscle metabolic adaptation, rather than to enable direct comparisons between different cell lines. Under stressful conditions, exposure to conditioned media derived from the hepatic compartment led to reduced ATP generation, increased oxidative stress, and reduced cellular viability. ROS levels decreased, while markers of cellular energy metabolism showed partial recovery following Supplement treatment. Although ATP and ROS measurements provide valuable insights into cellular bioenergetics, they are indirect indicators of mitochondrial function and should be interpreted in the context of the overall metabolic profile. A shift toward a more favourable metabolic phenotype is also suggested by changes in cytokine and myokine profiles, characterised by decreased levels of pro-inflammatory mediators and altered levels of adaptive myokines, including IL-6 and irisin. These molecules are known to play a central role in linking energy metabolism, mitochondrial function, and inflammatory responses [40,41]. Although IL-6 can act as a classic pro-inflammatory cytokine under chronic stress, muscle-derived IL-6 also functions as an energy-sensing myokine that promotes metabolic flexibility. Given the concurrent reduction in pro-inflammatory markers and the upregulation of irisin, the observed IL-6 elevation in our model reflects an adaptive metabolic response rather than a pro-inflammatory signal. An increase in cellular energy efficiency and metabolic flexibility is further supported by the restoration of glycogen levels, normalisation of calcium and magnesium fluxes, and reduction in lactate production. These findings should be interpreted as indicative of improved cellular adaptation to metabolic stress rather than a direct enhancement of muscle function.

These parameters are commonly associated with metabolic adaptation and energetic balance in skeletal muscle physiology [42,43]. The modulation of AMPK, p38, and ERK signalling pathways further supports the involvement of key molecular networks regulating cellular responses to oxidative and energetic stress. These pathways are known to coordinate adaptive and stress-related processes, including autophagy, inflammation, and metabolic remodelling [44,45]. Overall, the results suggest that the Supplement modulates interconnected pathways across biological compartments, including those involved in autophagy, cellular bioenergetics, oxidative stress, and inflammation. Given the formulation’s multi-component nature, the observed biological effects cannot be attributed to any single constituent. Rather, they reflect the biological activity of the complete formulation evaluated under the present experimental conditions. The relative contribution of each constituent and the presence of additive or synergistic interactions remain to be established in future studies specifically designed for this purpose. Given that the biological activity was evaluated using the complete formulation, the present study does not allow attribution of the observed effects to individual constituents, including low-abundance compounds such as spermidine, nor does it allow discrimination between the contribution of single compounds and that of the overall phytocomplex. Consequently, further studies employing isolated compounds or fractionated extracts are required to investigate this aspect. Evaluation of the complete formulation provides information on its overall biological activity but does not permit attribution of the observed effects to specific constituents or interactions among them.

This study has some limitations that should be considered. First, while useful for mechanistic insights, an in vitro model cannot fully recapitulate the complexity of in vivo physiological conditions, which involve multiple organs, systemic regulation, and immune responses. In addition, while several markers of autophagy and metabolic regulation were evaluated, complementary approaches, including genetic modulation studies and additional in vivo validation, would further strengthen the mechanistic interpretation of the results. Another limitation is inherent to the sequential conditioned-medium approach itself. Although this experimental strategy enables controlled investigation of compartment-to-compartment biological responses, it cannot completely exclude the contribution of residual soluble molecules transferred between compartments. Consequently, the present model should be interpreted as a mechanistic platform reproducing selected aspects of intercompartmental communication rather than a complete physiological gut–liver–muscle axis. In addition, a limitation of the present work is that only the complete formulation was investigated. Accordingly, the present study should be interpreted as an evaluation of the biological activity of the complete chemically characterised formulation and not as evidence supporting the activity of any individual constituent. Finally, further studies are needed to confirm these findings in vivo and to better clarify the underlying biological mechanisms.

## 4. Materials and Methods

### 4.1. Chemical Analysis of Natural Extracts

#### 4.1.1. Polydatin HPLC Determination

The content of polydatin in *Fallopia japonica* and the Supplement was determined by high-performance liquid chromatography (HPLC; Agilent, Santa Clara, CA, USA) using a C18 column and a UV detector (Infinite 200 Pro MPlex, Tecan, Männedorf, Switzerland) set to 303 nm. The mobile phase was composed of a mixture of acetonitrile and aqueous phosphoric acid (25:75), with a flow rate of 1.0 mL/min and a temperature of 30 °C. The Supplement containing polydatin was prepared in diluted ethanol to a concentration of approximately 0.2 mg/mL and 10 µL was used to quantify polydatin concentration in the Supplement by comparing the peak areas of the standard solution.

#### 4.1.2. Spermidine HPLC Determination

Spermidine was determined using an extraction and chromatographic analysis procedure for polyamines. In summary, wheat germ extract and the Supplement sample were dissolved in water and 1 mL of this solution was mixed with 100 µL of benzoyl chloride and 5 mL 2 mM NaOH. Then, the mixture was left to react at 37 °C water bath for 20 min and, at the end of incubation, 5 mL NaCl was added to the solution. After the solution was centrifuged, 1 mL was taken and dissolved in 1 mL of methanol. Quantitative analysis was performed using HPLC (Agilent, Santa Clara, CA, USA) with UV detection (Infinite 200 Pro MPlex, Tecan, Männedorf, Switzerland) and a mobile phase composed of methanol and water solution (70:30 *v*/*v*; flow rate of 1.0 mL/min). The detection was performed at a wavelength of 230 nm and the concentration of the spermidine in the Supplement was calculated based on calibration curves obtained from a standard solution.

#### 4.1.3. Ascorbic Acid TLC Determination

Ascorbic acid content was measured using thin-layer chromatography (TLC). Analyses were conducted on silica gel plates as the stationary phase, suitable for the separation of polar compounds such as ascorbic acid. *Rosa canina* extract and Supplement samples were dissolved with 60% ethanol solution and centrifuged; the supernatant was filtered prior to application onto the TLC plate and chromatographic separation was achieved using an appropriate mobile phase (acetic acid, acetone, methanol and toluene; 5:5:20:70 *V*/*V*/*V*/*V*). Following chromatographic development, the TLC plates were analysed under UV illumination at 254 nm. The chromatographic bands corresponding to ascorbic acid were identified by comparison with authenticated reference standards analysed under identical chromatographic conditions. Band intensity was subsequently evaluated by densitometric analysis, allowing semi-quantitative estimation of the relative ascorbic acid content. The reported percentages therefore represent the estimated recovery of ascorbic acid relative to the corresponding reference standard and do not derive from direct quantitative HPLC determination.

#### 4.1.4. 5-HTP Determination

5-HTP content in *Griffonia simplicifolia* and in the Supplement was determined by HPLC (Agilent, Santa Clara, CA, USA) and UV detection (Infinite 200 Pro MPlex, Tecan, Männedorf, Switzerland). Analyses were performed using a C18 column (250 mm × 4.6 mm, 5 µm), maintained at 30 °C. The mobile phase consisted of methanol and water (7:93, *V*/*V*), delivered at a flow rate of 1.0 mL/min and detection was carried out at 276 nm, with an injection volume of 10 µL.

*Griffonia simplicifolia* and the Supplement were weighed, dissolved in water, and subjected to ultrasonic extraction for 30 min. After cooling, the solution was brought to volume and filtered through a 0.45 µm membrane prior to analysis. The reference solution was prepared in the same manner.

Chromatographic analysis was performed after system stabilisation, and the reference solution was injected in triplicate to verify system precision. Sample solutions were then analysed under the same conditions, and chromatograms were recorded.

Quantification of 5-HTP was performed using the external standard method by comparing peak areas of the sample and standard solutions. The final content was calculated taking into account sample weight, dilution volume, standard purity, and loss on drying, and expressed as the mean of duplicate determinations.

### 4.2. Agent Preparation

A commercially available multi-component nutraceutical formulation was evaluated in the present study. The complete qualitative and quantitative composition is reported below. The formulation was kindly provided by Salugea (Falciano, San Marino). Each daily dose of the Supplement contained 500 mg of wheat germ extract (*Triticum aestivum* L.) rich in spermidine (with 1 mg of spermidine), 150 mg of *Rosa canina* L. pseudofruit dry extract standardised to 70% vitamin C (with 105 mg of Vitamin C), 100 mg of *Griffonia simplicifolia* Baill. seed dry extract standardised to 25% 5-hydroxytryptophan (with 25 mg of 5-HTP), 51 mg of *Fallopia japonica* (Houtt.) Ronse Decr. root dry extract standardised to 98% polydatin (with 50 mg of polydatin), 50 mg of PANMOL^®^ NADH micro (with 5 mg of NADH), 5 mg of zinc (from Zn-bisglycinate), and 41 µg of selenium (from Se-L-methionine). For the experiments, capsules were opened, and the powder was reconstituted in Dulbecco’s Modified Eagle’s Medium (DMEM, Merck Life Science, Rome, Italy), supplemented with 50 IU/mL penicillin–streptomycin (Merck Life Science, Rome, Italy), and 2 mM L-glutamine solution (Merck Life Science, Rome, Italy). The solution was then diluted to achieve a final concentration of 1:2000 in each well and specific concentrations are reported in Table A1 in Appendix A.

To simulate metabolic and inflammatory stress, cells were treated with a Free Fatty Acid (FFA) medium consisting of a 1:2 mixture of palmitic and oleic acid (both at final concentration of 0.25 mM; Merck Life Science, Milan, Italy), alongside Lipopolysaccharide (LPS; Merck Life Science, Milan, Italy) prepared as a 100x stock solution to achieve a final concentration of 100 ng/mL in Caco-2 cells [26]; the resulting supernatant was subsequently collected and used to treat HepG2 cells to investigate the intestinal-hepatic axis. Within this model, CQ (Merck Life Science, Milan, Italy) was added at a final concentration of 50 µM as a pharmacological inhibitor of the autophagic flux [46].

Furthermore, caffeine was prepared as a stock solution and diluted to a final concentration of 2.5 mM to treat C2C12 cells and evaluate its effects on skeletal muscle metabolism [47].

### 4.3. Cell Cultures

For the study, a standard model of the intestinal epithelial barrier using Caco-2 cells obtained from the American Type Culture Collection (ATCC, Manassas, VA, USA) was employed [48]. This cell line was maintained in an incubator at 37 °C with 5% CO_2_ and cultured in Advanced Dulbecco’s Modified Eagle Medium (Adv-DMEM; GIBCO, Thermo Fisher Scientific, Waltham, MA, USA), containing 2 mM l-glutamine (Merck Life Science, Rome, Italy), 1% penicillin-streptomycin (Merck Life Science, Rome, Italy) and 10% FBS (Merck Life Science, Rome, Italy) at 37 °C in a 5% CO_2_ incubator [49]. The cells were seeded onto 6.5 mm Transwell^®^ inserts (Corning, Corning, NY, USA) to establish an in vitro intestinal barrier model, with the culture medium replaced every other day on both the apical and basolateral sides until full maturation was achieved. Barrier integrity was assessed using an EVOM3™ (World Precision Instruments, Sarasota, FL, USA) to measure TEER. Measurements were recorded every two days for 21 days, until TEER values reached the threshold of ≥400 Ω·cm^2^ [50].

Human epithelial hepatocellular carcinoma HepG2 cells (ATCC, Manassas, VA, USA) were cultured in Adv-DMEM (GIBCO, Thermo Fisher Scientific, Waltham, MA, USA), supplemented with 10% FBS (Merck Life Science, Rome, Italy), 2 mM L-glutamine (Merck Life Science, Rome, Italy), and 1% penicillin–streptomycin (Merck Life Science, Rome, Italy) at 37 °C and 5% CO_2_. Cells used in the experiments were at 90–95% confluence [51].

Murine C2C12 myoblasts (ATCC, Manassas, VA, USA) were cultured in DMEM supplemented with 10% FBS (Merck Life Science, Rome, Italy), 100 U/mL penicillin/streptomycin, and maintained in an incubator at 37 °C with 5% CO_2_. Cells were maintained at a confluence of 40–70% to preserve their proliferative state and avoid induction of myogenic differentiation [47]. For differentiation, C2C12 myoblasts were cultured in DMEM supplemented with 2% horse serum for 7 days, with medium replaced every 48 h, until multinucleated myotubes were formed. Differentiated myotubes were then exposed to conditioned medium collected from the hepatic compartment according to the experimental protocol.

### 4.4. Experimental Protocol

**Figure 8:**
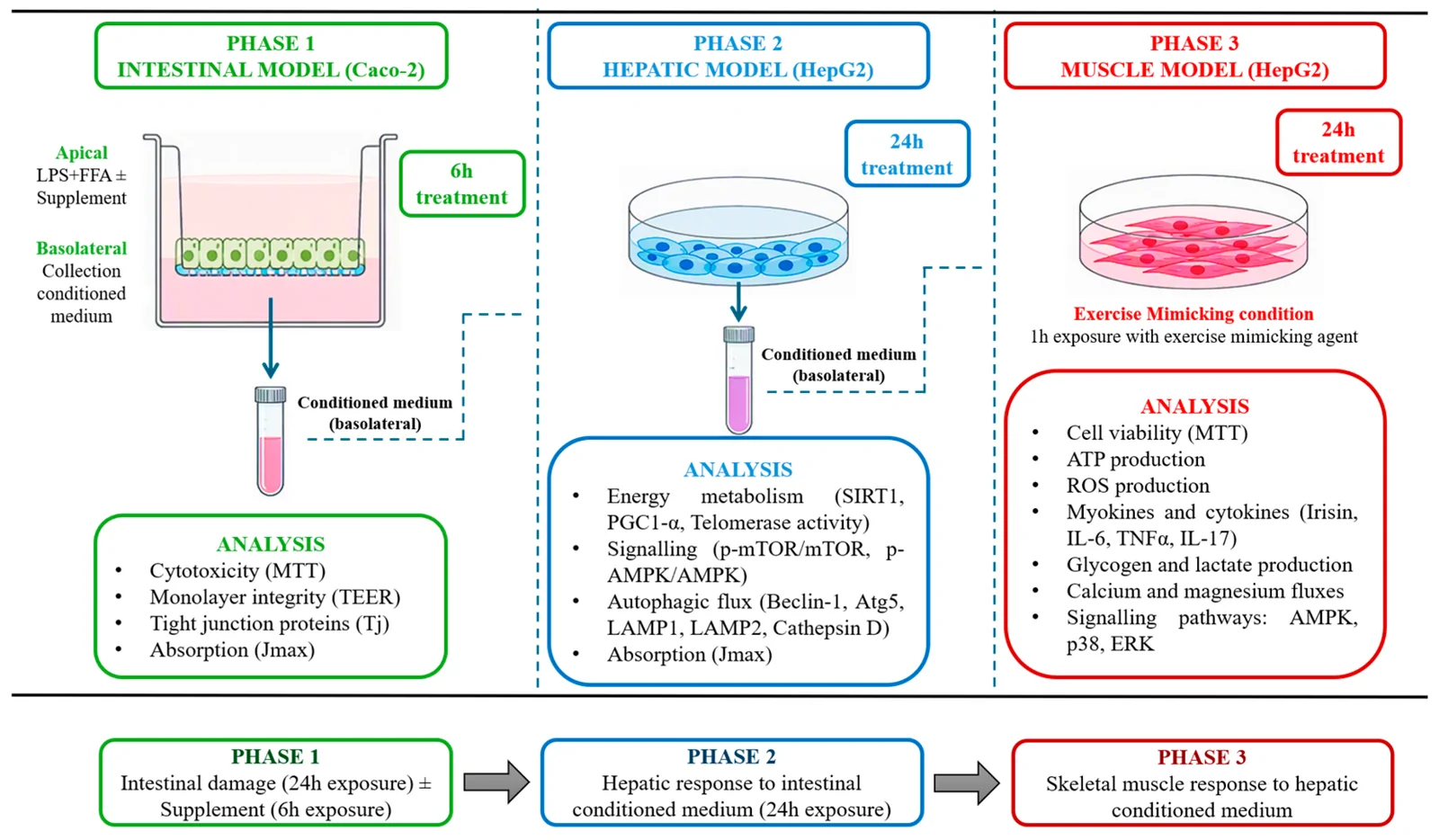
Schematic representation of the experimental design. The study was conducted in three sequential phases to evaluate the effects of the nutraceutical supplement in LPS+FFA-induced damage conditions. In Phase 1, Caco-2 cells grown on Transwell^®^ inserts were exposed to LPS+FFA, either with or without the supplement, for between one and six hours, to perform the relevant analyses. The conditioned culture medium collected from the basolateral compartment was then transferred to HepG2 cells in Phase 2 to evaluate energy metabolism, proteostasis, autophagic flux and signalling pathways (SIRT1, PGC-1α, mTOR and AMPK). Finally, the conditioned culture medium from the HepG2 cells was transferred to the differentiated C2C12 myotubes (Phase 3) to study cell viability, bioenergetics, oxidative stress, the release of cytokines and myokines, glycogen metabolism, ion fluxes and the signalling pathways involved in muscle adaptation.

The experiments were divided into three phases to investigate the effects of the nutraceutical Supplement against a damage-induced condition, starting from the intestinal barrier and progressing to hepatic and muscle models (Figure 8).

Phase 1. To simulate a metabolic syndrome- or NAFLD-like environment, Caco-2 cells were exposed to a medium containing LPS and FFA for 24 h before treatment [26]. A cytotoxicity study was conducted on these challenged intestinal cells to assess the Supplement’s safety at the intestinal level, evaluating cell viability via the 3-(4,5-dimethylthiazol-2-yl)-2,5-diphenyltetrazolium bromide (MTT) assay and monolayer integrity via TEER and tight junction (TJ) protein levels. Furthermore, after confirming the absence of relevant cytotoxic effects, paracellular permeability profile was assessed using a fluorescein probe to determine the maximum permeation rate (Jmax) across the intestinal monolayer. To perform these analyses, an in vitro intestinal barrier model was established using a Transwell^®^ system, in which the apical compartment was treated with LPS+FFA and the Supplement.

Phase 2. In this second phase, a hepatic model was developed using HepG2 cells treated with the conditioned medium derived from the damaged/treated Caco-2 basolateral compartment. This phase evaluated the effects of the Supplement on hepatic energy metabolism and proteostasis under damage-induced conditions, assessing SIRT1 (Sirtuin 1), PGC-1α, and telomerase activity, as well as the signalling of phosphorylated mammalian target of rapamycin (p-mTOR)/mTOR and phosphorylated AMP-activated protein kinase (p-AMPK)/AMPK. A detailed analysis of the autophagic flux was performed by measuring Beclin-1, Atg5 (with HAT as a supplementary factor), and the lysosomal markers LAMP1, LAMP2, and Cathepsin D. To accurately assess the autophagic turnover, cells were also treated with CQ as a pharmacological inhibitor. Specifically, LC3-I and LC3-II levels were quantified using a dedicated assay kit, while p62/SQSTM1 levels were analysed via Western blot to determine the p62/β-actin ratio relative to control. This combined approach enabled assessment of protein accumulation and flux blockade induced by the different experimental treatments.

Phase 3. In the final phase, conditioned medium from the hepatic compartment was collected and transferred to differentiated C2C12 myotubes to assess the systemic impact of the initial damage and subsequent treatment. In this stage, cell viability (MTT), cellular bioenergetics, assessed by adenosine triphosphate (ATP) production, and reactive oxygen species (ROS) generation (cytochrome C reduction) were evaluated. To further characterise the muscle response under these stressed conditions, the release of myokines and cytokines, including Irisin, Interleukin-6 (IL-6), Tumour Necrosis Factor α (TNF-α), and Interleukin-17 (IL-17), was quantified using a specific ELISA kit (FineTest, Wuhan, China), along with intracellular glycogen content, lactate production, and calcium and magnesium fluxes. The activation of key signalling pathways, including AMPK, p38, and ERK, was also analysed.

In all phases, before stimulations, all cells were synchronised overnight in DMEM without phenol red and FBS (Merck Life Science, Rome, Italy), supplemented with 1% penicillin/streptomycin, 2 mM L-glutamine, and 1 mM sodium pyruvate, and maintained at 37 °C in a humidified atmosphere with 5% CO_2_.

The sequential transfer of conditioned media was intentionally adopted to investigate biological responses generated by the preceding cellular compartment under controlled experimental conditions. This approach was designed as a mechanistic model of intercompartmental communication rather than as a complete reconstruction of the physiological gut–liver–muscle axis. As recognised for other conditioned-medium-based multi-organ models, this experimental strategy enables compartment-specific optimisation and investigation of sequential biological responses while not fully reproducing the complexity of in vivo inter-organ communication.

### 4.5. Conditioned Medium Collection and Transfer Procedure

To reproduce some aspects of gut-liver-muscle communication, conditioned media were sequentially transferred between the different cell compartments. At the end of the respective treatment periods, conditioned media were collected after 6 h from the Caco-2 compartment and after 24 h from the HepG2 compartment. The collected media was transferred directly to the next cell model using a volume of 200 µL for the liver model and 500 µL for the muscle model and was used immediately without further filtration, concentration, preservation, or chemical modification.

In the gut-liver phase, 200 µL of conditioned media was collected from the basolateral compartment of the Caco-2 model grown on a Transwell^®^ and transferred to the HepG2 cells. The conditioned media replaced 100% of the recipient cell culture medium. Subsequently, 600 µL of conditioned medium from HepG2 cells was collected after 24 h and transferred to differentiated C2C12 myotubes. Medium replacement procedures were performed identically for all experimental groups.

To ensure comparability between the different experimental conditions, the injury groups received conditioned media generated in the presence of all stimuli required by the protocol, including LPS (100 ng/mL), FFA (1:2 mixture of palmitic and oleic acid), high glucose (30 mM), and caffeine (2.5 mM), when applicable to the specific experimental phase. Conversely, the control groups received the corresponding control medium transferred sequentially between the different compartments according to the same experimental procedure.

Because the model is based on the sequential transfer of conditioned medium, the contribution of residual molecules in the transferred medium cannot be completely ruled out. Therefore, the system reproduces some aspects of gut-liver-muscle communication without directly representing the entire physiological axis in vivo.

### 4.6. Intestinal In Vitro Model

A Transwell^®^-based in vitro intestinal barrier model was established to evaluate compound permeability across the intestinal epithelium, in line with FDA and EMA recommendations [52,53] and following previously validated methodologies [54]. This system is widely used to investigate intestinal paracellular permeability of orally administered compounds. Caco-2 cells were seeded onto Transwell^®^ inserts and cultured in complete medium in both apical and basolateral compartments for 21 days, with medium replaced every other day to allow full differentiation and formation of a functional monolayer [55]. TEER was continuously monitored using an EVOM3™ voltohmmeter equipped with STX2 electrodes (World Precision Instruments, Sarasota, FL, USA) to assess monolayer integrity and tight junction formation. Only inserts with TEER values above 400 Ω·cm^2^ at day 21 were considered suitable for permeability studies [50]. Prior to stimulation, the apical compartment medium was adjusted to pH 6.5, reflecting the physiological conditions of the intestinal lumen, while the basolateral compartment was maintained at pH 7.4 to mimic systemic circulation [56]. Cells were then exposed to the test substances for 1–6 h. Paracellular permeability was evaluated using a 0.04% fluorescein tracer (Santa Cruz, CA, USA) at each time point [48]. Caco-2 monolayers were incubated with the tracer for 40 min at 37 °C, and fluorescein transport was quantified by measuring fluorescence with a spectrophotometer (Infinite 200 Pro MPlex, Tecan, Männedorf, Switzerland) at an excitation wavelength of 490 nm and an emission wavelength of 514 nm.

The permeation rate (J) was calculated according to the Michaelis–Menten equation:

J = Jmax [C]/(Kt + [C])

where

- Jmax represents the maximum permeation rate;
- [C] is the initial fluorescein concentration;
- Kt is the Michaelis–Menten constant.

Results were expressed as mean ± standard deviation (SD, %) and included negative controls without cells to account for passive diffusion across the Transwell^®^ membrane.

Fluorescein was used as a paracellular permeability marker to characterise the permeability properties of the intestinal epithelial monolayer and to calculate Jmax. This assay does not directly quantify the transport of the nutraceutical formulation or its individual constituents but provides a well-established functional assessment of epithelial permeability and absorptive behaviour in accordance with validated Caco-2 methodologies and current regulatory recommendations [56,57,58,59,60].

### 4.7. Hepatic Model

The hepatic phase utilised HepG2 cells seeded in the basolateral compartment of a Transwell^®^ system to investigate the metabolic effects of the Supplement after intestinal processing. To reproduce selected features of NAFLD-like metabolic stress, the hepatocytes were subjected to metabolic stress induced by a high-glucose environment (30 mM glucose) [61]. The cells were then stimulated with the conditioned medium collected from the basolateral chamber of the previous Caco-2 model, containing conditioned medium collected from the basolateral compartment following intestinal exposure.

### 4.8. Cell Viability Analysis

The MTT cell viability assay was performed according to a standardised protocol to assess potential cytotoxic effects [47]. Absorbance was measured at 570 nm, with background correction at 690 nm, using a spectrophotometer (Infinite 200 Pro MPlex, Tecan, Männedorf, Switzerland). Cell viability was calculated relative to untreated control cells (set as 0% baseline). Data were reported as mean ± SD from five independent experiments, each performed in triplicate.

### 4.9. Tight Junction Analysis

Following the manufacturer’s instructions, Caco-2 lysates were analysed for the quantification of occludin protein levels using the Human Occludin (OCLN) ELISA kit (MyBiosource, San Diego, CA, USA), for claudin-1 levels using the (LCC) ELISA kit (Cusabio Technology LLC, Houston, TX, USA), and for ZO-1 activity using the Human Tight Junction Protein 1 (TJP1) ELISA kit (MyBiosource, San Diego, CA, USA) [48]. Absorbance at 450 nm was measured using a spectrophotometer (Infinite 200 Pro-MPlex, Tecan). Data were compared to the standard curve, which ranged from 0 to 1000 pg/mL for ZO-1 and claudin-1, and from 0 to 1500 pg/mL for occludin. Results are expressed as a percentage (%) relative to the control (baseline, set as 0) from five independent experiments performed in triplicate.

### 4.10. PGC-1α ELISA Kit

PGC-1α levels were analysed using a PGC-1α ELISA kit (Antibodies, Stockholm, Sweden) [62]. Samples were measured at 450 nm using a microplate reader (Infinite 200 Pro MPlex, Tecan, Männedorf, Switzerland). Concentrations were expressed in ng/mL relative to a standard curve (ranging from 0 to 10 ng/mL), and results were presented as a percentage (%) of the control.

### 4.11. Telomerase Levels

Telomerase levels were measured using a Human Telomerase ELISA kit (Invitrogen, Waltham, MA, USA) [63]. Samples were read at 450 nm using a microplate reader (Infinite 200 Pro MPlex, Tecan, Männedorf, Switzerland). Telomerase concentrations were calculated using a standard curve spanning 0.16 to 10 ng/mL and expressed in ng/mL. Results were reported as a percentage (%) relative to the control.

### 4.12. SIRT-1 ELISA Kit

SIRT-1 levels were analysed using a SIRT1 ELISA kit (Thermo Fisher Scientific™, Waltham, MA, USA) [64]. Samples were measured at 450 nm with a microplate reader (Infinite 200 Pro MPlex, Tecan, Männedorf, Switzerland). Concentrations were expressed in ng/mL relative to a standard curve (ranging from 1.23 to 300 ng/mL), and results were presented as a percentage (%) of the control.

### 4.13. LC3β, Beclin-1, and Atg5 ELISA Kits

LC3β, Beclin-1, and Atg5 levels were measured using Human Autophagy-Related Protein LC3β (LC3β), Human BECN1 (Beclin-1) [65], and Human ATG5 (Autophagy Protein 5) [66] ELISA kits (all from FineTest, Wuhan, China). Samples were read at 450 nm using a microplate reader (Infinite 200 Pro MPlex, Tecan, Männedorf, Switzerland). Protein concentrations were calculated from standard curves and expressed as ng/mL (LC3β: 20–4500 ng/mL; Beclin-1: 0.156–10 ng/mL; Atg5: 0.313–20 ng/mL). Results were expressed as a percentage (%) relative to the control.

### 4.14. HAT ELISA Kit

HAT levels were measured using an HAT Colourimetric Cell-Based ELISA Kit (Boster Biological Technology, Pleasanton, CA, USA) [67]. Samples were read at 450 nm using a microplate reader (Infinite 200 Pro MPlex, Tecan, Männedorf, Switzerland). Results were expressed as a percentage (%) relative to the control.

### 4.15. Western Blot

Following standard protocols, the HepG2 cells were lysed on ice using Complete Tablet Buffer (Roche, Basel, Switzerland) supplemented with 1 mM phenylmethanesulfonylfluoride (PMSF), 2 mM sodium orthovanadate (Na_3_VO_4_), phosphatase inhibitor cocktail (1:50), and protease inhibitor cocktail (1:200) [62]. Protein extracts (35 µg) were separated by 8–15% SDS-PAGE using Precision Plus Protein All Blue Prestained Protein Standards (Bio-Rad Laboratories, Hercules, CA, USA, Cat. No. 1610373) as molecular weight markers, and transferred onto PVDF membranes (GE Healthcare Europe GmbH, Milan, Italy). Membranes were incubated overnight at 4 °C with primary antibodies. The antibodies against p-AMPKα1/2, AMPKα1/2, p-mTOR, mTOR, SQSTM1/p62 (1:500; Santa Cruz Biotechnology, Santa Cruz, CA, USA) and the loading control β-actin (1:4000; Merck Life Science, Rome, Italy) were mouse monoclonal. To detect the autophagic markers LC3-I and LC3-II, a rabbit polyclonal antibody against MAP1LC3A (1:500; Merck Life Science, Rome, Italy) was used. Secondary antibodies were chosen according to the host species of the primary antibody: HRP-conjugated anti-mouse IgG (1:5000; Merck Life Science, Rome, Italy) was used for all Santa Cruz and β-actin antibodies, while an HRP-conjugated anti-rabbit IgG (1:5000; Merck Life Science, Rome, Italy) was employed for the LC3 analysis. Immunoreactive bands were visualised using an enhanced chemiluminescence detection system. Band intensities were quantified by densitometric analysis, normalised to β-actin or to the corresponding total protein, as appropriate, and expressed relative to untreated control cells. Results are expressed as mean (%) ± SD, relative to untreated control cells (0% line).

### 4.16. LAMP1 and LAMP2 Elisa Kits

LAMP1 and LAMP2 levels were measured using Human Lysosomal Associated Membrane Protein 1 (LAMP1) [48] and Human Lysosomal Associated Membrane Protein 2 (LAMP2) ELISA kits (MyBioSource, San Diego, CA, USA) [68]. Samples were read at 450 nm using a microplate reader (Infinite 200 Pro MPlex, Tecan, Männedorf, Switzerland). Protein concentrations were calculated from standard curves and expressed as ng/mL (LAMP1: 0.625–40 ng/mL; LAMP2: 0.156–10 ng/mL). Results were expressed as a percentage (%) relative to the control.

### 4.17. Cathepsin D Elisa Kit

Cathepsin D levels were measured using a Human Cathepsin D (CTSD) ELISA kit (MyBioSource, San Diego, CA, USA) [69]. Samples were read at 450 nm using a microplate reader (Infinite 200 Pro MPlex, Tecan, Männedorf, Switzerland). Protein concentrations were calculated from standard curves and expressed as ng/mL (Cathepsin D: 3.12–200 ng/mL). Results were expressed as a percentage (%) relative to the control.

### 4.18. ROS Evaluation

ROS production was quantified using a cytochrome C reduction-based assay (Merck Life Science, Milan, Italy). Absorbance at 550 nm was measured in culture supernatants using a spectrophotometer (Infinite 200 Pro MPlex, Tecan). O_2_^−^ levels were expressed as mean ± SD (%) of nanomoles of reduced cytochrome C per microgram of protein relative to the control [70].

### 4.19. ATP Production

At the end of each stimulation, cells were immediately treated with the components of an ATP assay kit (Calbiochem, San Diego, CA, USA). Luminescence was measured 1 min after adding the ATP-monitoring enzyme using a spectrophotometer (Infinite 200 Pro MPlex, Tecan). ATP levels were calculated as µmol ATP/g protein and expressed as mean ± SD (nanomoles, nmol) per well [47].

### 4.20. Cytokine and Myokine Quantification by ELISA

TNF-α, IL-17, and IL-6 levels were quantified using commercially available ELISA kits (FineTest, Wuhan, China) [71,72] according to the manufacturers’ instructions. Samples were read at 450 nm using a microplate reader (Infinite 200 Pro MPlex, Tecan, Männedorf, Switzerland). TNF-α concentrations were calculated using a standard curve spanning 0 to 6000 pg/mL and expressed in pg/mL, with results reported as a percentage (%) relative to the control. IL-17 levels were determined by comparing sample optical density (OD) values to a standard curve (1.6–100 pg/mL). Five independent experiments were performed in duplicate, and results were expressed as mean ± SD (%) relative to the control. IL-6 concentrations in cell culture supernatants were calculated using a standard curve spanning 0.078 to 5 pg/mL and expressed as pg/mL, with results reported as a percentage (%) relative to the control. Irisin levels were measured using a Human Irisin ELISA kit (FineTest, Wuhan, China) [73]. Absorbance was read at 450 nm using a microplate reader (Infinite 200 Pro MPlex, Tecan). Irisin concentrations were calculated using a standard curve spanning 1.56 to 100 ng/mL and expressed as a percentage (%) relative to the control.

### 4.21. Lactate and Glycogen Assays

Lactate and glycogen levels in C2C12 cells were quantified using commercially available assay kits [47] following the manufacturer’s instructions. Lactate was measured in cell lysates, with lactate and pyruvate normalised to total protein and expressed as a percentage (%) relative to the control. Glycogen synthesis was assessed by centrifuging samples at 13,000 rpm for 5 min, hydrolysing glycogen to glucose using an OxiRed probe (BioVision/Abcam, Cambridge, UK), and measuring absorbance at 570 nm (Infinite 200 Pro MPlex, Tecan). Glycogen concentration was calculated as C = Ay/Sv, where Ay is the glycogen (mg) from the standard curve, and Sv is the sample volume (mL); results were expressed relative to the control.

### 4.22. p38, ERK, and AMPK ELISA Kits

Phosphorylated p38 MAPK, ERK/MAPK, and AMPK levels were quantified using commercially available ELISA kits (for Phosphorylated p38 MAPK: Abcam, Cambridge, UK; for ERK/MAPK and AMPK: Thermo Fisher, Milan, Italy) [62,74] following the manufacturer’s instructions. Cell lysates were used for all assays, and absorbance was measured at 450 nm using a microplate reader (Infinite 200 Pro MPlex, Tecan, Männedorf, Switzerland). Phospho-p38 and ERK results were expressed as a percentage (%) relative to the control. Phosphorylated AMPK levels were quantified from a standard curve (0–50 U/mL) and expressed both in U/mL and as a percentage (%) relative to the control.

### 4.23. Calcium/Magnesium Fluxes

Intracellular Mg^2+^ and calcium fluxes were assessed using the fluorescent dye Mag-fura-2-AM (Furaptra, Biotium, Fremont, CA, USA) and Fura-2AM (Merck Life Sciences, Roma, Italia), as previously described [75]. Briefly, for Mg^2+^ fluxes cells were incubated in Mg^2+^-free Hanks’ balanced salt solution (Thermo Fisher Scientific, Waltham, MA, USA) supplemented with 10 mM glucose, 20 mM HEPES/Tris (pH 7.4), 1.3 mM CaCl_2_, and 5 μM Mag-fura-2-AM at 37 °C for 30 min. For Ca^2+^, cells were incubated for 30 min at 37 °C with 5 μM Fura-2AM in Ca^2+^-free physiological saline solution (PSS; 1.5 mM KCl, 10 mM HEPES, 10 mM D-glucose, 2 mM L-glutamine, pH 7.4) under gentle shaking and protected from light. Fluorescence of Mag-fura-2-loaded and Fura-2AM cells was monitored with excitation of 340/380 nm and emission of 510 nm using a fluorescence spectrophotometer (Infinite 200 Pro MPlex, Tecan) with an exposure time of 100 ms. The data were expressed as a percentage change relative to the control cells and reported as the mean ± SD.

### 4.24. Statistical Analysis

Statistical analyses were performed using GraphPad Prism (version 9.5.0). Data obtained from experiments involving a single independent variable were analysed using one-way ANOVA followed by Bonferroni’s post hoc test. Experiments involving two independent variables, including time-course studies and treatment × inhibitor experiments, were analysed using two-way ANOVA followed by Bonferroni’s post hoc test for multiple comparisons. Results are presented as mean ± standard deviation (SD) from at least five independent biological experiments. Statistical significance was accepted at *p* < 0.05. Statistical analyses were performed using the original experimental values before to graphical normalisation.

## 5. Conclusions

In summary, the present study suggests that a multi-component nutraceutical Supplement may modulate pathways involved in autophagy and metabolic regulation within an integrated intestinal–hepatic–muscle axis model. Within the limitations of an in vitro preclinical framework, the Supplement was associated with preservation of intestinal barrier integrity, modulation of hepatic nutrient-sensing pathways, and improvement in markers of skeletal muscle metabolic adaptation under conditions of metabolic stress. These findings support further investigation of chemically characterised multi-component nutraceutical formulations as modulators of interconnected pathways involved in autophagy-related processes, oxidative stress, inflammation and metabolic adaptation. Given the complexity of the formulation and the experimental model employed, further in vivo and mechanistic studies are required to confirm these observations and clarify the underlying biological pathways. Overall, this work provides a preliminary framework for future investigations exploring integrated nutraceutical strategies in metabolic dysfunction.

## Figures and Tables

**Table 1:** Quantitation of a selection of bioactive markers in individual extracts. Polydatin content in *Fallopia japonica* extract, spermidine content in wheat germ extract, vitamin C content in *Rosa canina* extract, and 5-HTP content in *Griffonia simplicifolia* extract are reported in the table. Values are reported as mean ± SD from five independently prepared analytical replicates, each analysed in triplicate.

Natural Extract	Bioactive Constituent	Content (%)
*Fallopia japonica*	Polydatin	98 ± 0.97
Wheat germ extract	Spermidine	0.2 ± 0.01
*Rosa canina*	Vitamin C	70 ± 0.54
*Griffonia simplicifolia*	5-HTP	25 ± 0.48

**Table 2:** Analytical recovery of selected bioactive marker compounds in the final formulation. Percentages represent the analytical recovery of each marker relative to its expected content after incorporation into the formulation and do not indicate the percentage composition of the total formulation. Values are reported as mean ± SD from five independently prepared analytical replicates, each analysed in triplicate.

Natural Extract	Bioactive Constituent	Analytical Recovery (%)
*Fallopia japonica*	Polydatin	97.7 ± 1.02
Wheat germ extract	Spermidine	0.18 ± 0.01
*Rosa canina*	Vitamin C	About 68–72%
*Griffonia simplicifolia*	5-HTP	24.8 ± 0.65

## Data Availability

The data supporting the findings of this study are available from the corresponding author upon reasonable request. All relevant data are included within the article.

## Abbreviations

The following abbreviations are used in this manuscript:

5-HTP	5-hydroxytryptophan
Adv-DMEM	Advanced Dulbecco’s Modified Eagle Medium
AMPK	AMP-activated protein kinase
ATCC	American Type Culture Collection
ATG	Autophagy-related gene proteins
ATP	Adenosine triphosphate
BECN1	Beclin 1
CQ	Chloroquine
CTSD	Cathepsin D
DMEM	Dulbecco’s Modified Eagle’s medium
FBS	Foetal bovine serum
FFAs	Free fatty acids
HAT	Histone acetyltransferase
IL-6	Interleukin-6
IL-17	Interleukin-17
LAMP1	Lysosomal Associated Membrane Protein 1
LAMP2	Lysosomal Associated Membrane Protein 2
LC3	Microtubule-associated protein 1 light chain 3
LPS	Lipopolysaccharide
mTOR	Mammalian Target of Rapamycin
mTORC1	mTOR complex 1
MTT	3-(4,5-dimethylthiazol-2-yl)-2,5-diphenyltetrazolium bromide
NADH	Nicotinamide adenine dinucleotide
NAFLD	Non-alcoholic fatty liver disease
OCLN	Occludin
OD	Optical density
p-AMPK	Phosphorylated AMP-activated protein kinase
PGC-1α	Peroxisome proliferator-activated receptor gamma coactivator 1-alpha
PMSF	Phenylmethanesulfonylfluoride
p-mTOR	Phosphorylated Mammalian Target of Rapamycin
ROS	Reactive oxygen species
SD	Standard deviation
SIRT1	Sirtuin 1
TEER	Transepithelial electrical resistance
TJ	Tight junction
TJP1	Tight Junction Protein 1
TNF-α	Tumour Necrosis Factor α

## Appendix A

**Table A1:** Initial dosages and diluted dosages administered to cells for all components of the final formulation tested.

Individual Component	Initial Dosage	Diluted Dosage
wheat germ extract	500 mg/mL	250 µg/mL
*Rosa canina* L.	150 mg/mL	75 µg/mL
*Griffonia simplicifolia*	100 mg/mL	50 µg/mL
*Fallopia japonica*	51 mg/mL	26 µg/mL
PANMOL^®^ NADH	50 mg/mL	25 µg/mL
Zinc	5 mg/mL	3 µg/mL
Selenium	41 µg/mL	0.021 µg/mL
